# Simulation-Based Resilience Quantification of an Indoor Ultrasound Localization System in the Presence of Disruptions

**DOI:** 10.3390/s21196332

**Published:** 2021-09-22

**Authors:** Aishvarya Kumar Jain, Dominik Jan Schott, Hermann Scheithauer, Ivo Häring, Fabian Höflinger, Georg Fischer, Emanuël A. P. Habets, Patrick Gelhausen, Christian Schindelhauer, Stefan Johann Rupitsch

**Affiliations:** 1 Fraunhofer Institute for High-Speed Dynamics, Ernst-Mach-Institute (EMI), 79588 Efringen-Kirchen, Germany; ivo.haering@emi.fraunhofer.de (I.H.); Fabian.Hoeflinger@emi.fraunhofer.de (F.H.); Georg.Fischer@emi.fraunhofer.de (G.F.); patrick.gelhausen@fernuni-hagen.de (P.G.); 2 Department of Microsystem Engineering (IMTEK), University of Freiburg, 79110 Freiburg, Germany; dominik.jan.schott@imtek.uni-freiburg.de (D.J.S.); stefan.rupitsch@imtek.uni-freiburg.de (S.J.R.); 3 Hahn-Schickard, 78052 Villingen-Schwenningen, Germany; Hermann.Scheithauer@hahn-schickard.de; 4 International Audio Laboratories Erlangen, University of Erlangen-Nuremberg, 91058 Erlangen, Germany; emanuel.habets@audiolabs-erlangen.de; 5 Department of Computer Science (IIF), University of Freiburg, 79110 Freiburg, Germany; schindel@informatik.uni-freiburg.de

**Keywords:** indoor localization, simulation, time difference of arrival, disruption, technical resilience, localization accuracy, ultrasound, cross correlation, loss function, resilience engineering

## Abstract

Time difference of arrival (TDOA) based indoor ultrasound localization systems are prone to multiple disruptions and demand reliable, and resilient position accuracy during operation. In this challenging context, a missing link to evaluate the performance of such systems is a simulation approach to test their robustness in the presence of disruptions. This approach cannot only replace experiments in early phases of development but could also be used to study susceptibility, robustness, response, and recovery in case of disruptions. The paper presents a simulation framework for a TDOA-based indoor ultrasound localization system and ways to introduce different types of disruptions. This framework can be used to test the performance of TDOA-based localization algorithms in the presence of disruptions. Resilience quantification results are presented for representative disruptions. Based on these quantities, it is found that localization with arc-tangent cost function is approximately 30% more resilient than the linear cost function. The simulation approach is shown to apply to resilience engineering and can be used to increase the efficiency and quality of indoor localization methods.

## 1. Introduction

Acoustic indoor localization systems, with applications in logistics, consumer electronics, health care, security, and catastrophe management, are required to be precise and reliable. Furthermore, they should be resilient to expected disruptions [1,2,3,4]. Physical testing of these systems during the development phase can incur a huge amount of time, increasing the development and other overhead costs. Simulating the behavior of these systems can reduce time and money. As the performance of these systems is correlated to the operational environment in which they are employed, it is essential to simulate the behavior of the environment. Two of the most important environmental factors that affect the performance of acoustic indoor localization are the geometry of the room and the absence or presence of disruptions. Disruption refers to any process that hinders the normal functionality of the localization system, for example, noise in the environment or malfunctioning receivers. A framework capable of simulating different environments and disruptions can be used to simulate the behavior of indoor localization systems and evaluate the performance in different environments and with different disruptions.

The main objective of the paper is to present a systematic methodology to test TDOA-based indoor ultrasound localization systems in a simulation environment before entering the production phase and during product improvement cycles. The aim is to quantify and visualize the localization accuracy and resilience in the presence and absence of disruptions. To achieve this objective, we propose an end-to-end simulation and evaluation framework, which includes a method to perform acoustic source localization and a method to evaluate the system’s performance and measure its resilience. The proposed framework can be used to detect the strengths and weaknesses of localization algorithms during the development and testing phase. As an example, we used the proposed framework to test one of the existing iterative TDOA-based localization algorithms, but the same methodology can be applied to test other TDOA-based algorithms. The simulation of the indoor ultrasound propagation is performed for a shoe-box room and can be extended to more complex room geometries. We also demonstrate how this framework can be used to induce multiple disruptions and how the performance of the localization system can be quantified and compared in the presence of such disruptions. In particular, we focused on some basic disruptions due to noise and barriers as well as receiver malfunction. This framework uses an informed method for system improvement in terms of localization accuracy and resilience, which is demonstrated by an improved functionality when using an arc-tangent loss function instead of a linear loss function.

### 1.1. Related Work

Acoustic indoor localization is a widely studied subject, see [5] and the references therein. Different mechanisms, such as time of flight, doppler effect, and phase shift, can be employed. In the present work, we use TDOAs, which are computed based on time of flight information. The location of a source can then be computed from the TDOAs (see, for example, [6]), which remains a subject of ongoing research. Comuniello et al. [7], for example, recently proposed a best linear unbiased estimator for TDOA-based localization, which is more robust against multipath propagation.

Different frequency ranges can be employed for acoustic indoor localization. The BeepBeep localization system [8] uses the frequency range from 1 kHz up to 20 kHz. This frequency range also offers the possibility to use smartphones, without extra hardware, for localization [9,10]. Using a commercial off-the-shelf component, one can push the frequency range outside the audible domain, as the ASSIST system [11] does.

Previous studies related to indoor localization have adopted a simulation-based approach to assessing the system’s performance. In the radio frequency domain, Sapumohotti et al. [12], for example, proposed a simulation test-bed for WiFi-based localization systems. Their motivation was to reduce the development and testing cost by providing a simulated environment to test the WiFi-based localization systems and algorithms. A similar approach was described by Alhammadi et al. [13], simulating the received signal strength in an indoor environment by considering a multiwall path-loss model. They validated the simulated data using an experimental test bench and showed that simulated data can be used to develop and evaluate high-accuracy localization systems.

To produce representative simulation data, we need to model the propagation of sound waves. In enclosed rooms, the propagation can be simulated using geometric methods (e.g., image-source [14,15,16,17] and ray-acoustic methods [18,19,20,21]), or wave-based methods (e.g., finite element [22] or boundary element methods [23]). When considering ultrasound waves, we can assume that wavelength is much smaller than the dimensions of the enclosed room and the size of most obstacles. In this work, a ray-acoustic method is adopted as it can take into account solid obstacles and exhibits a lower computational complexity compared to wave-based methods.

The operational performance of localization systems vastly depends on the operating environment and the disruptions. The effect of noise on the performance of acoustic indoor localization systems has been studied, for example, in [24]. In practice, the operational performance of a localization system depends on the combination of disruptions and not only noise. Therefore, it is important to study the behavior of acoustic indoor localization systems in the presence of several expected disruptions and measure the resilience of the system against these disruptions. However, the domain of simulation including these disruptions and resilience quantification is relatively unexplored.

### 1.2. Structure of This Paper

The remainder of the paper is organized as follows. In Section 2, we discuss the employed acoustic simulation method and explain how the disruptions are modeled. In Section 3, the TDOA-based localization system under test is described. First, we describe the process of computing TDOAs from the generated RIRs and estimating the acoustic source position from the TDOAs. In Section 4, we discuss how to quantify the resilience of a technical system and correlate it with the simulated indoor localization system presented in this paper. In Section 5, we present the results. First, we investigate the localization with individual disruptions and compare the performance of arc-tangent and linear loss functions. Secondly, we evaluate the performance in the presence of multiple disruptions. Finally, we analyze the time-dependent resilience curves, which are used to calculate the resilience of the simulated indoor localization system. In Section 6, we use data collected from real experiments to validate our simulation results. Section 7 presents the conclusion and major findings.

## 2. Proposed Simulation Framework

### 2.1. Acoustic Simulation

In this work, we simulated the indoor ultrasound propagation for all the source and receiver pairs and all frequencies using the ray acoustics method. Each propagation path is modeled by a room impulse response (RIR). A single acoustic source was placed at a random position in the room at a pre-defined height $z_{s}$. In addition, the *N* receivers were placed randomly in the room at a fixed height $z_{r}$. The frequency range for the simulation was limited to 19–20 kHz.

### 2.2. Disruption Modeling

We have included three kinds of disruptions in the simulation framework, namely, barrier in the vicinity of the acoustic source, noise in the environment, and malfunction of the receivers. The disruptions are added individually and also in combination (of two disruptions).

To introduce a barrier into the simulation, a new set of acoustic simulations is performed for each barrier orientation to generate new RIRs specific to the orientation of the barrier.

To introduce noise into the system, the zero-mean Gaussian noise [25] with standard deviation σ is directly added to the reverberant signal g(t). The noise is quantified relative to the original signal by comparing the signal-to-noise ratio (SNR) [26]

(1) $${\mathrm{SNR}}_{dB}=10\;\log_{10}\left(\frac{P_{S}}{P_{N}}\right),$$

where $P_{S}=T^{-1}\int_{0}^{T}g{\left(t\right)}^{2}dt$ is the power of the signal g(t) and $P_{N}=\sigma^{2}$ is the power of the generated noise signal and *T* is the time period of the signal.

To introduce the effect induced by receiver malfunction, only a subset of receiver instances are used from all the receiver instances. This subset is selected at random from the total set of receiver instances.

## 3. Localization System under Test

The system under test is a TDOA-based indoor ultrasound localization system. In such systems, the clocks of the acoustic source are not synchronized with the clocks of acoustic receivers, but clocks of all the acoustic receivers are synchronized together. In such a case, the time of flight of the signal cannot be estimated and thus they resort to the estimation of TDOAs for the localization [27]. The formalization described in the following subsections is used for the implementation of the indoor localization system used in this paper.

### 3.1. TDOA Estimation

For the ultrasound localization system used in this study, linear sine chirps of the form

(2) $$s\left(t\right)=\sin\left(2\;\pi \left(f_{0}+\frac{f_{1}-f_{0}}{2\;T}t\right)t\right),$$

are used, because of their high signal-to-noise ratio (SNR) [11]. To generate reverberant signals at each receiver, discrete signal convolution [28] is performed between simulated RIR and the input chirp signal from Equation (2).

The accuracy of the localization system depends on the extraction of correct timestamps, that is, the times when the receivers respectively receive the direct signals, which are termed time of arrival (TOA) timestamps. The TOAs can be extracted through signal cross-correlation between the reverberant signal at each receiver and the original signal [29]. The TOA timestamp is the time where the cross-correlation peaks. Because of reflection, there could be multiple peaks, but the most dominant first peak is created by the direct signal as the direct line of sight between the receiver and source is the shortest path for the signal to travel. If there is an obstruction between the direct line of sight then the dominant peak is generated by the reflected signal. This potentially introduces errors in the source position estimation, but such anomalies could be handled by the loss functions and the minimization method introduced in the Section 3.2. Once TOA timestamps are generated for all the receivers, TDOA timestamps for all receiver source pairs are generated [30],

(3) $$\begin{array}{cc} {\mathrm{TDOA}}_{ij} & ={\mathrm{TOA}}_{i}-{\mathrm{TOA}}_{j},\\ i, & j\in 1,2,\ldots ,N,\\ i & \neq j. \end{array}$$

For a system with *N* receivers, in total $\left(\begin{array}{c} N\\ 2 \end{array}\right)$ TDOAs are generated. From the generated TDOAs, the difference between the distance from the source to receiver i and receiver j referred to as the distance difference is calculated using

(4) $${\Delta d}_{ij}=c\;\cdot \;{\mathrm{TDOA}}_{ij},$$

where *c* is the speed of sound in air.

### 3.2. Position Estimation

The position of the source is calculated using the non-linear least square method with slight modifications [31]. The objective function for the non-linear least square problem is

(5) $$\begin{array}{cc} f\left(q_{s};\left\{{\Delta d}_{ij}\right\};\left\{q_{i}\right\}\right) & =\sum_{i,j}^{N}\rho \left({\left(\frac{{\Delta d}_{ij}-h_{ij}\left(q_{s}\right)}{S}\right)}^{2}\right),\\ \mathrm{where}\;q & =\left[x,y,z\right], \end{array}$$

where $q_{s}$ is the source position, $\left\{\Delta d_{ij}\right\}$ is the measured distance difference between receivers in Equation (4), *S* is the scaling factor, and $\left\{q_{i}\right\}$ is a set with the known receiver positions, with equal and same height $z_{r}$

(6) $$\forall i:z_{i}=z_{r}.$$

The function $h_{ij}\left(q\right)$ is the expression for the distance difference between receiver i and receiver j.

(7) $$h_{ij}\left(q\right)=∥q_{i}-q∥-∥q_{j}-q∥.$$

The function ρ represents the loss function. In this paper, the linear and arc-tangent loss functions [32] are used, i.e.,

(8) $$\rho_{lin}\left(a\right)=a,$$

(9) $$\rho_{at}\left(a\right)=\arctan\left(a\right).$$

For given receiver locations $\left\{q_{i}\right\}$, the non-linear least square estimate of the source coordinates ${\hat{q}}_{s}$ using Equation (5) [31] is

(10) $${\hat{q}}_{s}=\arg\;\min_{x,y}f\left(q_{s};\left\{{\Delta d}_{ij}\right\};\left\{q_{i}\right\}\right),$$

which is estimated using an iterative trust-region reflective algorithm [33] as implemented in the Python routine *scipy.optimize.least_square* (2019). With the rather explicit notation introduced in Equations (3)–(7), Equation (10) is generated and can be used to estimate the source position. Furthermore, other standard approaches to solving the TDOA problem using matrix inversion and iterative matrix equation solvers are documented in the work of Jain [27].

The loss function introduced in Equation (9) can lead to significant improvement in the localization accuracy when disruptions are present in the surrounding environment. The arc-tangent loss function helps to eliminate outliers, which appear when localization is done in the presence of disruptions [34]. The effect is illustrated by comparing the results obtained using the linear and arc-tangent loss functions.

To plot “time-dependent resilience curves”, a weighted smoothing with a window length of $N_{w}$ and weight vector $w_{m}$ is performed on predicted source position vector at *n*th time index.

(11) $${\tilde{q}}_{s}\left(n\right)=\frac{1}{\sum_{m=0}^{N_{w}-1}w(m)}\sum_{m=0}^{N_{w}-1}w\left(m\right)\;q_{s}\left(n-m\right).$$

## 4. Resilience Quantification

Resilience quantification of a system is crucial in identifying the vulnerability of the system to various kinds of disruptions. Such disruptions can put the system’s functionality to risk. Resilience estimates the functionality of the system in the presence of all representative disruptions. In the case of technical systems, resilience quantification is based on a mathematical formulation of the system’s performance. In the current context, it is represented by the localization error, calculated in meters [35]. In the approach shown below, we have extended the resilience methodology to apply in the context of indoor localization systems.

Technical resilience is based on a scalar product of two different vectors: a and b, which are probabilities of occurrence of each event and cost function of each event, respectively. Both a and b are dimensionless non-negative vectors. Given a and b the resilience of the system is quantified as,

(12) $$\mathrm{Rsl}=\exp(-a^{T}\;b).$$

**Figure 1:**
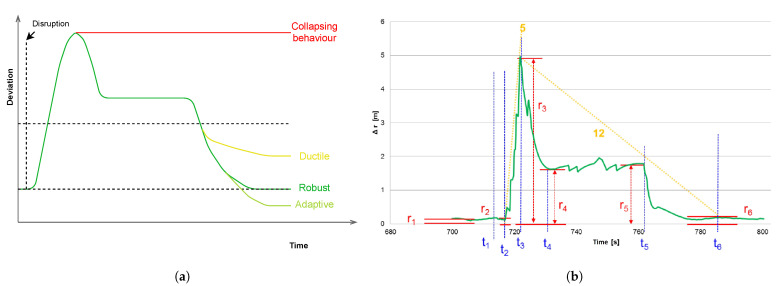
(**a**) Different types of performance behavior i.e., collapsing, ductile, robust, and adaptive behavior of a system. (**b**) Performance behavior of a sample technical system along with corresponding system parameters.

Figure 1a shows performance behavior for a system. The bright green curve represents the system’s response to a rare event, “adaptive behavior”, if the disruption has improved the system’s performance. The yellow curve is the “ductile behavior”, if the performance mildly deteriorates, i.e., the performance stabilizes under a tolerance level. Last, the red curve is the worst case, “collapsing behavior” where the system function breaks down. The dark green curve is the standard case of ”robust behavior”, where the system reaches the same performance level as before the disruption.

Figure 1b represents the robust behavior type of a technical system. The green curve denotes the localization error in meters as a function of time. The representation is termed as “Normally down”. Figure 1b shows six different errors (in red) which are as follows [36,37]:

- *r*_1_: Performance before disruption,
- *r*_2_: Performance after delay time,
- *r*_3_: Maximum performance loss,
- *r*_4_: Temporary performance level, during response phase while disruption continues,
- *r*_5_: Performance during the end of disruption, beginning of recovery phase,
- *r*_6_: Performance after total recovery.

These errors occur at different times, quantified in seconds (blue markers in Figure 1b) as follows:

- *t*_1_: Time of sudden disruption,
- *t*_2_: End of delay time, begin of protection phase,
- *t*_3_: Time to reach maximum performance loss,
- *t*_4_: Time when response-reaction occurs,
- *t*_5_: End of disruption, beginning of recovery,
- *t*_6_: End of complete recovery, when the pre-disruption state is restored.

The yellow indicators in Figure 1b describe the rate of rise and fall of the performance loss from the ground state:

- “5”: rate $\left(r_{3}-r_{2}\right)/\left(t_{3}-t_{2}\right)$ describes the rate of maximum performance loss during “protect phase”,
- “12”: rate $\left(r_{6}-r_{3}\right)/\left(t_{6}-t_{3}\right)$ is the rate of bounce back to the pre-disruption state during “response and recover phase”.

**Figure 2:**
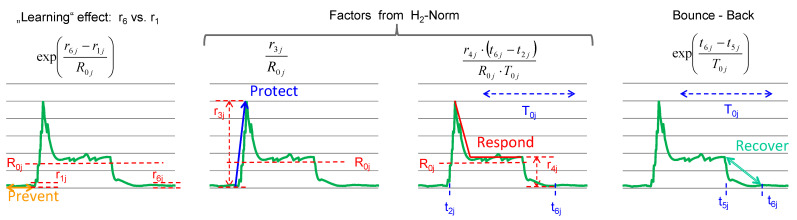
Different phases of a resilience curve showing “Prevent”, “Protect”, “Respond” and “Recover” phases along with the corresponding loss factor. “Bounce-back” effect refers to the recovery phase of the system.

Gross et al. [38] describe the optimization of system resilience as a structured $H_{2}$ norm optimization. It is an integral-quadratic performance criterion that quantifies the system’s behaviour after shock. The system behaviour type is like a PD-control. Comparing Figure 1a,b the system response is “robust type” between $t_{2}$ and $t_{5}$. Figure 2 shows the quantification of different phases of a resilience curve in terms of loss factor using the indicators described above (except the yellow indicators). The product of the four factors shown in Figure 2 is the “consequence ($b_{j}$)”, which is a component of the vector b in Equation (12),

(13) $$b_{\;j}=\exp\left[\frac{r_{6\;j}-r_{1\;j}}{R_{0\;j}}\right]\frac{r_{3\;j}\;r_{4\;j}\;\left(t_{6\;j}-t_{2\;j}\right)}{R_{0\;j}^{2}\;T_{0\;j}}\;\exp\left[\frac{t_{6\;j}-t_{5\;j}}{T_{0\;j}}\right].$$

The probability vector a has the component,

(14) $$a_{\;j}=\left[\frac{t_{6\;j}-t_{2\;j}}{T_{tot}}\right],$$

which is the quotient between the disruption duration and the total system lifetime ($T_{tot}$).

In Equation (13), the factors $r_{3j}r_{4j}\left(t_{6j}-t_{2j}\right)$ are an approximation of the quadratic integral. They are scaled by the “Norm performance ($R_{0j}$)” and “Characteristic time ($T_{0j}$)”. These factors only quantifies the “Protect” and “Respond” phases (Figure 2). A single disruption in a robust behaviour also have “Prevent” and “Recover” phases [39,40,41]. To accommodate these two phases in the resilience quantification, two additional exponential factors (Figure 2) are introduced.

Substitution of Equations (13) and (14) in the Equation (12) leads to the derivation of the resilience of a sample technical system [38] shown in Figure 1b as,

(15) $$\mathrm{Rsl}=\exp\left[-\sum_{j=1}^{H}\frac{t_{6\;j}-t_{2\;j}}{T_{tot}}\;\exp\left[\frac{r_{6\;j}-r_{1\;j}}{R_{0\;j}}\right]\;\frac{r_{3\;j}\;r_{4\;j}\;\left(t_{6\;j}-t_{2\;j}\right)}{R_{0\;j}^{2}\;T_{0\;j}}\;\exp\left[\frac{t_{6\;j}-t_{5\;j}}{T_{0\;j}}\right]\right],$$

where *H* refers to all the disruption events that can affect the functionality of the system and $T_{tot}$ represent total system’s lifetime.

The proposed methodology is applied to the simulated indoor localization system in Section 5. Equations (13) and (14) are used to calculate the elements of b and a respectively. The corresponding values are then used to estimate the resilience of the system.

## 5. Evaluation and Discussion

The following sections present the localization error plots for linear and arc-tangent cost functions and the resilience quantification of the localization system under test.

### 5.1. Setup and Disruptions

**Figure 3:**
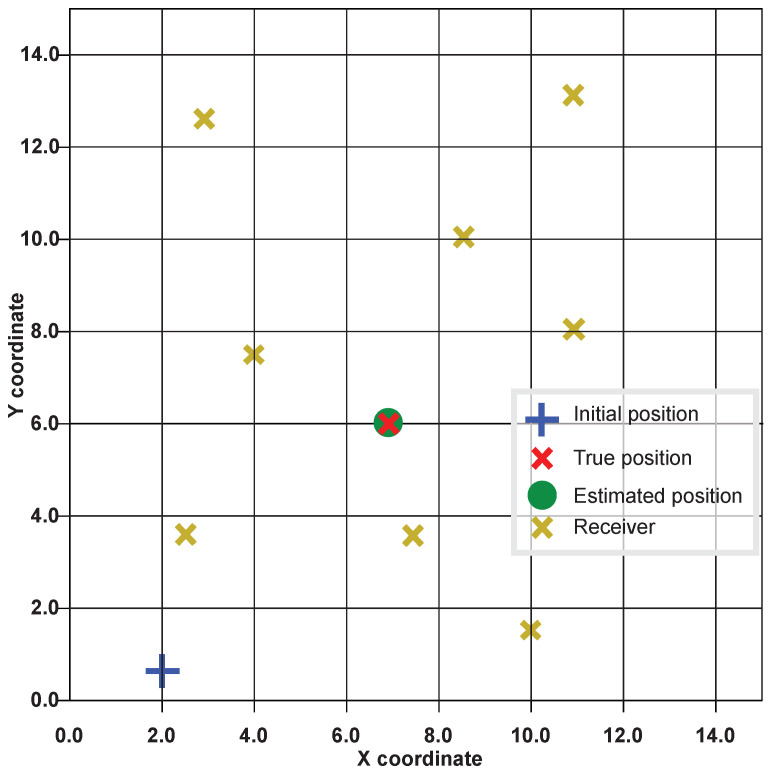
Acoustic simulation setup and implementation verification results showing the final estimated location (see green dot) when the algorithm is initialized with a random location (blue plus in left-bottom corner). In the absence of noise and barriers, and with all the eight receivers (yellow crosses) working, the observed Δ*r* = 0.0126 m (see the red cross for true source position). Room dimensions are 15 m × 15 m × 5 m. Receivers are placed at height $z_{r0}$ = 4.9 m and the source is located at height $z_{s0}$ = 1 m.

A shoe-box room of dimension 15 m × 15 m × 15 m which represent a standard office room, is used to demonstrate the proposed approach. The location of the source and receivers is shown in Figure 3, which is one of the many possible constellations. The constellation under test was chosen randomly while keeping in mind that the receivers are spread throughout the dimensions of the room. To simulate the room acoustics, we used the ray acoustics module of COMSOL (version 5.3a) [42], furthermore, we work under the assumption that the simulator software provides reliable and realistic results. It is important to mention that this approach can be used to test such a localization system within a much more complex room geometry and with different source-receiver constellations. The speed of sound is assumed to be equal to 343 m/s.

**Figure 4:**
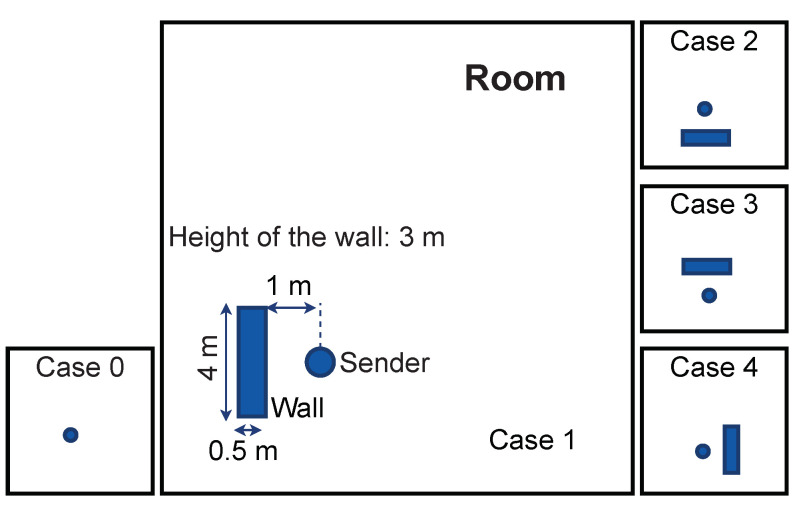
Different orientations of barrier relative to the position of the source representing different cases of barrier disruption.

For barrier as disruption, four different barrier orientations are used as shown in Figure 4.

For the simulations with increasing noise in the localization environment, μ=0 and σ increase in steps of 0.1 from 0 to 1. This corresponds to SNR_dB_ ranging from ∞ to −3 dB according to Equation (1). Although more simulations were performed with lower SNR_dB_, we observed that beyond −3 dB SNR, the extracted TOAs are not very accurate, resulting in a very high localization error.

For the simulation with a decreasing number of active receivers, the number of receivers is decreased from eight to three. We believe the upper bound of eight receivers is representative of a room of this dimension. Although, later on, we see the same localization accuracy can also be achieved with fewer receivers, in our study, we decided to use eight receivers.

The localization error plots show different disruption cases along the x-axis and the obtained Δr on the y-axis. For the first instance of each simulation, the source position is initialized at random and for later simulated instances, the initialization is done with the final localization estimation of the previous instance. The results are plotted as box plots (bar plot for the barrier as disruption). These curves indicate the robustness of the implemented localization system with respect to the disruptions and compare the performance of linear and arc-tangent loss functions. For each disruption case, 100 source instances (with the same source position) are evaluated and plotted using the box plots. Along with the instances, a mean (black diamond) and median (black horizontal line) of Δr is also plotted.

As described in Section 3.2, the arc-tangent loss function is expected to have a superior performance over the linear loss function in the presence of outliers. In addition to the localization error, the cumulative probability distribution of the predicted Δr is shown for the linear loss function.

Figure 3 shows the localization with linear loss function in the absence of disruptions (no barrier, no noise, and all receivers functioning) in case of random position initialization. The observed absolute distance between the true location and the estimated location is Δr = 0.0126 m.

Section 5.2 presents the result with only one single disruption. Section 5.3 presents the results with two disruptions present simultaneously, because, in reality, the combination of two or more disruptions is more likely.

### 5.2. Single Disruptions

**Figure 5:**
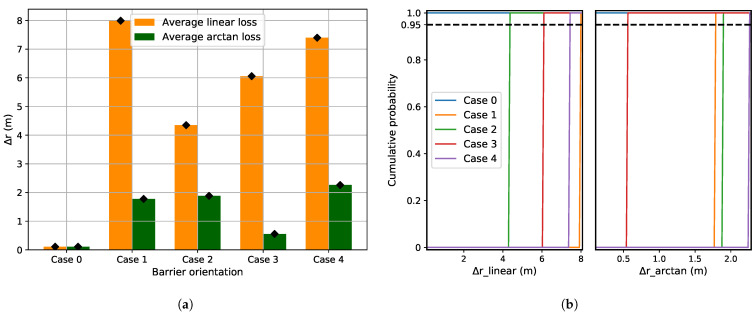
(**a**) Localization error for the barrier as disruption (with different barrier orientation as shown in Figure 4. For a static source, the location estimation is the same for a fixed barrier, thus generating the same localization error for each instance for a given case. (**b**) Cumulative distribution of Δ*r* for the linear and arc-tangent loss functions. As the location estimation is the same for a given barrier case, the cumulative function looks like a step function.

Figure 5a depicts the localization error in the presence of barriers for different cases of barrier orientation (Figure 4) estimated both for linear and arc-tangent loss functions. Figure 5b shows the cumulative distribution of predicted Δr plotted for both the loss functions. In the presence of a barrier, the direct line-of-sight paths between the sender and some of the receivers are obstructed. This induces outliers in the TDOAs and $\Delta d_{ij}$, and subsequently errors in the location estimated using Equation (10). The results in Figure 5a show that the arc-tangent loss function, which is more robust against outliers, results in smaller localization errors (Δr) than the linear loss function.

**Figure 6:**
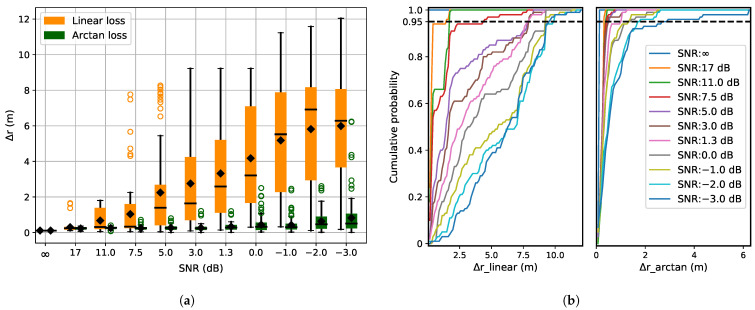
(**a**) Localization error with noise as disruption. The signal-to-noise ratio is given for each increasing noise level. (**b**) Cumulative distribution of Δ*r* for the linear and arc-tangent loss functions. As the noise in the system increases, the localization error shows a higher variance. The variance is comparatively less for the arc-tangent loss function.

Figure 6a depicts the localization error in the presence of noise, using box plots. Figure 6b shows the cumulative distribution of predicted Δr for both the loss functions. Introducing noise into the system introduces significant localization errors, which increases up to 6 m as the level of noise increases (Figure 6a). The error increases as the wrong peak is extracted from the cross-correlation function as described in Section 3.1. This leads to erroneous localization estimates as unreliable TOAs and hence TDOAs are used in Equation (10), resulting in outliers. Arc-tangent loss function being robust to outliers performs well in the presence of noise (Figure 6a).

**Figure 7:**
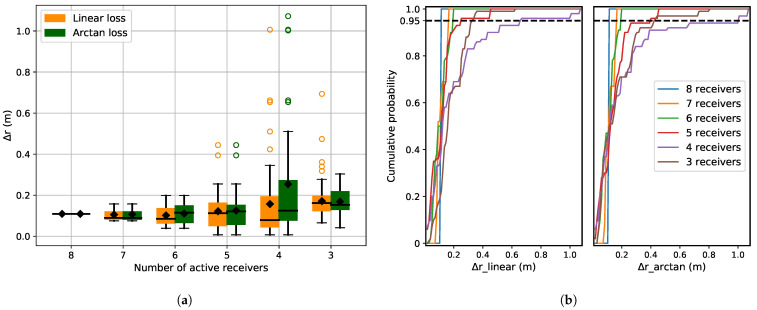
(**a**) Localization error for receiver malfunction as disruption for a system with 8 receivers. The different cases are generated by reducing the number of active receivers randomly from 8 to 2. (**b**) Cumulative distribution of Δ*r* for the linear and arc-tangent loss functions. The cumulative distribution is almost similar for both the loss functions.

Figure 7 shows localization error for receiver malfunction and cumulative distribution of predicted Δr plotted for both the loss functions. For receiver malfunction, reduced localization accuracy is visible when the number of active receivers reduces to perform the localization (Figure 7a). For this disruption, both arc-tangent and linear loss functions show comparable performance (Figure 7a). In this case, they have to cope with the system malfunction, which does not introduce any outliers, but rather reduces the number of TDOA values $d_{ij}$ available to solve the localization problem (Equation (10)).

It is also observed that even if the number of available receivers is only three, the observed Δr is small if the initialization of the position is close to the real position. The reason is that the objective function in Equation (5) is not concave and a good initialization ensures that it converges to a local minimum that is close to the global minimum. Another observation is the sudden increase in Δr for four receivers (Figure 7a), which is due to the maximum number of random combinations of receivers possible, i.e., 84.

The consolidated comparison between arc-tangent and linear loss is presented in Table 1. For barrier, the mean values of case 2 barrier are selected. For noise, the mean values at SNR = 5.0 dB are extracted. And for receiver malfunction, the mean values at 5 active receivers are used. These values are selected to have a representative amount of each disruption.

### 5.3. Multiple Disruptions

In this section, we combined two disruptions, which is a more realistic scenario, together and then evaluated the system’s performance. In all the evaluations, one disruption is kept constant and the other disruption is changed. Although there could be several more sets of two disruptions, we evaluated only three combinations in the present study. The first combination is constant barrier plus increasing noise. The second combination is constant noise and reducing the number of receivers. And the third combination is a constant number of available receivers and increasing noise. All the constant values are chosen at random to have a representative value of the disruption.

**Figure 8:**
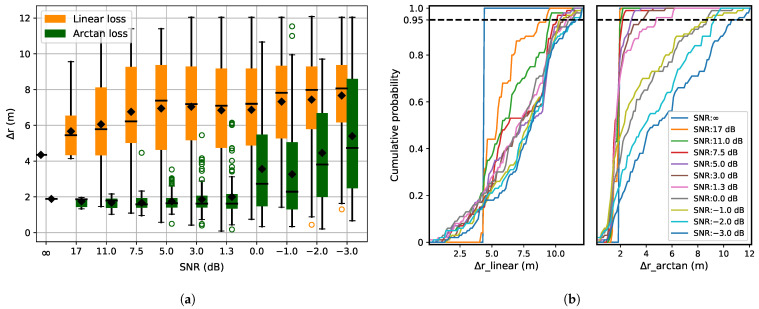
(**a**) Localization error with barriers and increasing noise. Barrier orientation is as in Case 2 of Figure 4. The white noise of zero means is varied with increasing standard deviation from 0 to 1 in steps of 0.1. (**b**) Cumulative distribution of Δ*r* for the linear and arc-tangent loss functions.

Figure 8a shows the localization error with barrier and increasing noise. The barrier is fixed (Case 2 of Figure 4) and noise is increasing as in Figure 6. Figure 8b shows the cumulative distribution of predicted Δr for both the loss functions. When comparing Figure 6a and Figure 8a, it becomes evident that even a small amount of noise has a strong negative influence on the localization accuracy if the localization is already disrupted with a barrier. In Figure 8a, the initial performance gap between the two loss functions is due to the superiority of the arc-tangent in the presence of a barrier. Further observation is also in agreement with what is observed in Figure 6a, i.e., that the arc-tangent has a better performance when the SNR decreases.

**Figure 9:**
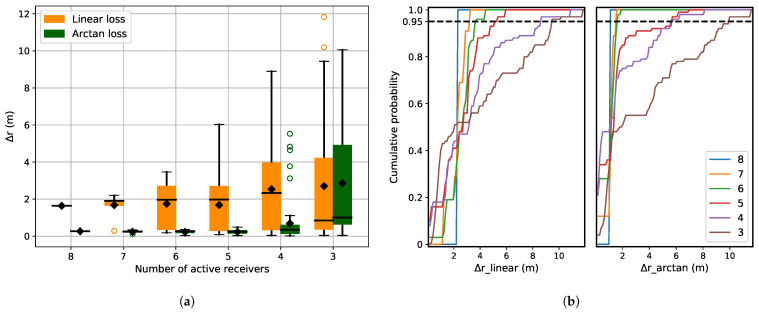
(**a**) Localization error plots with constant noise and reducing the number of active receivers. Throughout the simulation, white noise of *μ* = 0 and *σ* = 0.4 corresponding to the SNR = 5.0 dB is present. The numbers of active receivers are decreased from 8 to 2. (**b**) Cumulative distribution of Δ*r* for the linear and arc-tangent loss functions.

Figure 9a shows the effect of decreasing the receiver’s numbers for a given noise level. It is observed that the localization accuracy is further reduced if, in addition to the reduced number of active receivers, the noise is also present, as deduced when comparing Figure 7a and Figure 9a. It can be seen in Figure 9a that the arc-tangent shows superior performance compared to the linear loss function. The initial performance gap between arc-tangent and linear loss is due to the presence of a constant noise of mean μ=0 and standard deviation σ=0.4 corresponding to SNR = 5.0 dB. In this case, when reducing the number of active receivers, the arc-tangent keeps performing better. In the present case, the dominating performance of the arc-tangent is due to the hybrid nature of the disruptions, thus concluding that the arc-tangent shows superior performance for realistic disruption scenarios.

**Figure 10:**
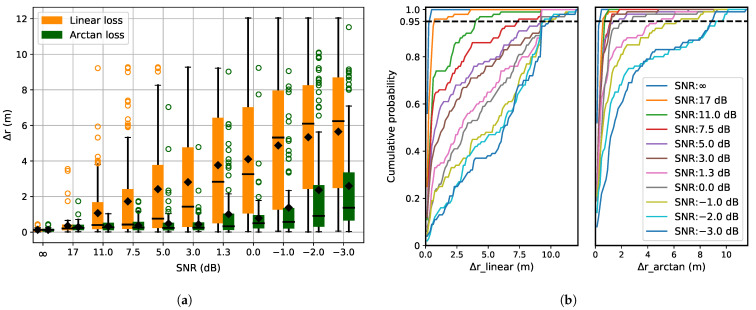
(**a**) Localization error with 5 active randomly selected receivers and increasing noise. (**b**) Cumulative distribution of Δ*r* for the linear and arc-tangent loss functionsn.

Figure 10 shows the effect of increasing noise when some receivers are already malfunctioning. When comparing the localization error with 5 receivers selected at random, the effect of noise on localization errors (Figure 10a) is found to be similar to the case of all receivers working (see Figure 6a). This is because the constant malfunctioning of three inactive receivers only causes minor localization errors. The absence of initial performance-gap between the two loss functions in comparison to Figure 8a and Figure 9a is because both arc-tangent and linear loss functions show a comparable performance for pure receiver malfunction (Figure 7a). As the noise is introduced in the system, the superiority of the arc-tangent loss function can be seen.

The consolidated comparison between arc-tangent and linear loss is presented in Table 2. For barrier plus noise, the mean values of barrier Case 2 and an SNR = 5.0 dB were selected. For noise plus receiver malfunction, the mean values at SNR = 5.0 dB with 5 active receivers were extracted. And for receiver malfunction plus noise, the mean values at 5 active receivers with SNR = 5.0 dB were used. The last two values represent the same scenario and thus they also have comparable localization errors. The difference appears because of different simulation runs. These values are selected to have a representative amount of each disruption.

### 5.4. Time-Dependent Resilience Curves for Linear Loss Function

In this section, the time-dependent resilience curves [4] are studied. The time-dependent resilience curve generation is based on the following modeling assumptions:

1. Consecutive chirps according to Equation (2) are spaced by 50 ms.
2. The computations are conducted with no delay between chirps.
3. Disruptions appear and disappear instantaneously, which is a simplification but very powerful for comparison of pure resilience response and recovery behavior. The duration of the disruptions is 5 s.
4. A uniform backward-looking moving average as in Equation (11) is plotted for $N_{w}=30$ instances in each case corresponding to a window of 1.5 s.
5. The first sender position initialization is random. Then, initialization is carried out using the estimation of the previous time step.

**Figure 11:**
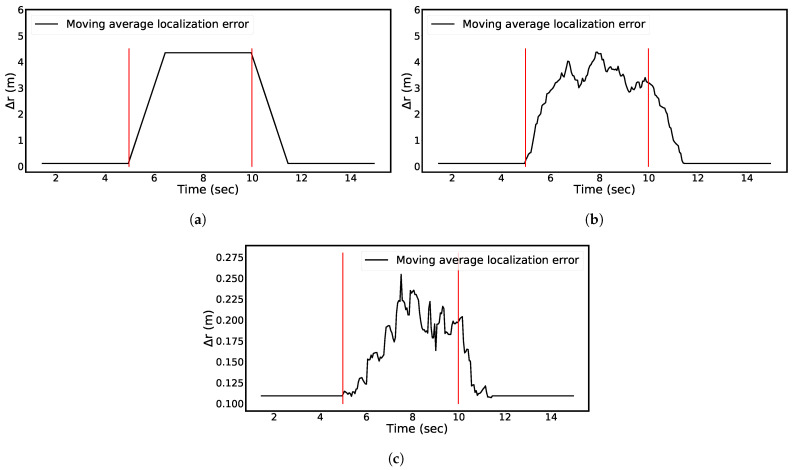
Time-dependent resilience curves with (**a**) barrier as disruption, (**b**) noise as disruption, and (**c**) receiver malfunction as disruption. The red lines mark start time *t*_1_ = 5 s and end time *t*_1_ = 10 s of the disruptions detailed in the main text.

Figure 11a assesses the time-dependent resilience behavior for a barrier of Case 2 shown in Figure 4 present for *T* = 5 s starting at *t*_1_ = 5 s and ending at *t*_2_ = 10 s. Figure 11b adds noise with μ=0, σ=0.4 which corresponds to SNR = 5 dB. Figure 11c shows the effect of switching off, at random, four receivers. In both cases, the disruption is introduced and removed at the same times as in Figure 9a.

The behavior seen in Figure 11a,b is as expected when inspecting Figure 5a and Figure 6a and taking the smoothing described under assumption 4into account. The time-dependent resilience response curve in Figure 11c shows that the smoothing has a rather small effect due to the large deviation in case of the random disruption present (Figure 7a). The latter type of disruption is physically not very likely when compared to the single loss of all but four receivers. However, all cases show benign degradation (e.g., there are no oscillations) and that the implemented algorithm recovers fast after all types of disruptions.

### 5.5. Resilience Quantification of Indoor Localization System

To apply “Rsl” (Equation (15)) to the simulations, the performance values $r_{j}$, $t_{j}$, performance tolerance $R_{0}$ and time tolerance $T_{0}$ are needed. Section 5.2 and Section 5.3 present the simulation results of the performance during a disruption, i.e., the values $r_{3\;j}$, $r_{4\;j}$. Additionally, Section 5.4 provides the information about the disruption time, i.e., $t_{2\;j}$, $t_{5\;j}$ and $t_{6\;j}$.

**Figure 12:**
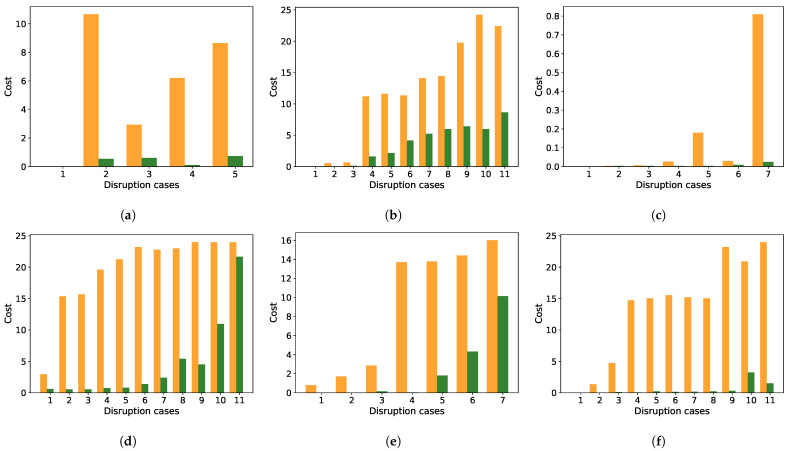
Cost function quantification and comparison between linear and arc-tangent loss function. Red color refers to the linear loss and green color refers to the arc-tangent loss. The diagrams are directly related to the simulation cases (**a**) barrier disruption, (**b**) noise disruption, (**c**) receiver malfunction, (**d**) barrier (case 2 of Figure 4) plus increasing noise, (**e**) fixed noise (SNR = 5 dB) plus receiver malfunction (reducing number of receivers), (**f**) receiver malfunction (three receivers are malfunctioning) plus increasing noise.

The simulations are distinct between two different objective functions for the nonlinear least-square optimization: first, a linear loss function, which is equal to the well-known linear least-square fit; and secondly, an arc-tangent loss function, where the arcus-tangent of a quadratic sum is minimized. In Figure 12, the red values quantify cost functions in the linear case, the green in arc-tangent fit. The values are dimensionless, because they are based on the performance tolerance level $R_{0}$ and the time tolerance duration $T_{0}$, and they are always positive.

Figure 12 shows that the cost functions in the case of arc-tangent fits are always smaller than in the linear case. So, it is clear that the arc-tangent fit is an improvement. Quantifying this in resilience calculus, we note that the cost functions are a part of resilience. Generally, Equation (15) is the e-function of a scalar product of $\vec{a}$ and $\vec{b}$ (Equation (12)). Since all components are positive, then: 0≤Rsl≤1, where 0 means complete destruction of a system. In the case of small *a*-values, the disruptions are rare, and Rsl is close to 1. If the disruptions are frequent, the scalar product grows, and Rsl decreases. It is a sum, i.e., one severe disruption can cause a low resilience, even if all other components are low. In such a case, the system is not well suitable for this kind of disruption, which can be caused by high *a* (happens often) or by high *b* (the system robustness against the disruption is bad) or both.

The probabilities of various disruptions in an indoor localization system are

1. Barrier: $a_{B}\;=\;0.0052$ in every case.
2. Noise: Sum of $a_{N}\;=\;0.02$ decreasing level.
3. Receiver malfunction: Sum of $a_{RM}\;=\;0.046$ decreasing level.

Combining the probabilities with the cost function result in resilience quantities for the simulated system that is displayed in the Table 3:

The results in Table 3 shows that the arc-tangent loss function is more resilient than the linear loss function.

## 6. Experimental Results

In this section, we experimentally verify whether the improvements observed when using the simulation environment can also be observed when using measured data. Previously, we found that the arc-tangent loss function provides more accurate location estimates than the linear loss function. Here, we verify whether this is true also for the data collected through the experiments. Although this section uses experimental data for the verification, it does not attempt to validate the simulations. Instead, we try to state that this simulation framework can be used to test acoustic indoor localization systems with a certain credibility.

**Figure 13:**
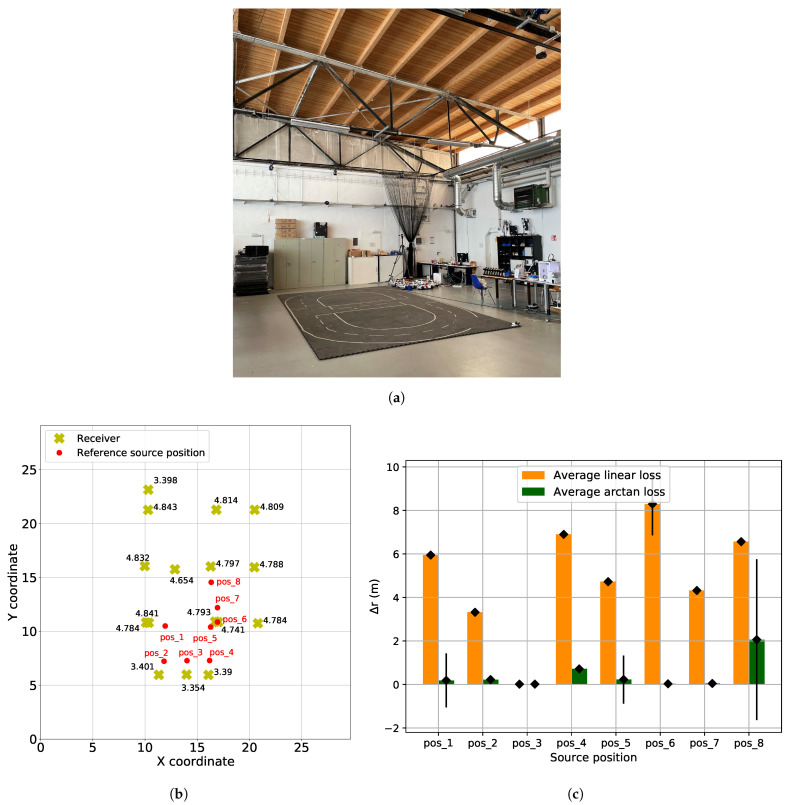
(**a**) Physical experimental site. Room dimensions are 29.1 m × 29.7 m. (**b**) Yellow crosses are the receiver with corresponding height written next to them. All the red dots represent the reference source positions on the ground. (**c**) Localization error for different source locations and loss functions.

The experimental site is shown in Figure 13a. The room is 29.1 m long and 29.7 m wide. For the first experiment, we installed 16 receivers in the room. The room dimension and corresponding arrangement of receivers are depicted in Figure 13a. For the experimental results, we repeated the static experiment for each source position and performed the localization using both arc-tangent and linear loss functions. For each source location, we ran the evaluation 500 times and plotted the results using the bar plots in Figure 13b.

As can be seen in Figure 13b, arc-tangent performs better than the linear loss function for different source locations. The reason being, arc-tangent is better in handling outliers (Section 3) i.e., the arc-tangent loss function is less sensitive to wrongly extracted TOAs than the linear loss function.

**Figure 14:**
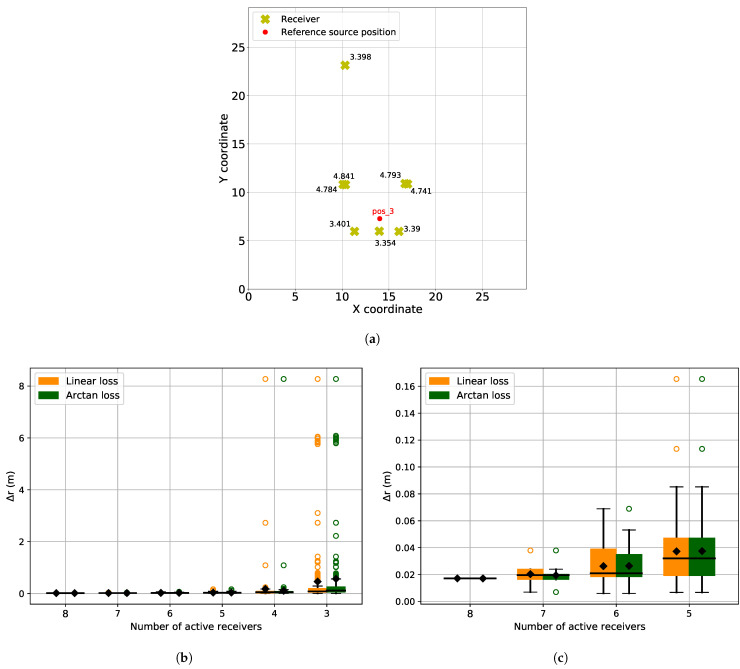
(**a**) Experimental setup: Yellow crosses are the receiver with corresponding heights written next to them. Only 8 receivers are selected with best SNR. The red dots represent the reference source position on the ground. (**b**) Localization error for different number of active receivers and loss functions, and (**c**) Snapshot of (**b**) for more than 4 receivers.

For the next experiment, we used one specific source position ($pos_{3}$) and selected 8 receivers out of 16 with the highest SNR. These receivers are shown in Figure 14a. In the experiment, we reduced the number of receivers from 8 to 3 and compared the localization accuracy using both arc-tangent and linear loss functions. For each case (number of receivers), we repeated the evaluation 500 times, where the active receivers were selected at random from the set of 8 receivers. The results are shown in Figure 14b. Figure 14c is the snapshot of Figure 14b to better visualize the performance of arc-tangent and linear when the number of receivers is more than 4.

In Figure 14b, we can see that for a given source, when the number of available receivers is reduced from 8 to 3, both arc-tangent and linear loss functions have the same performance. In this case, it is the receiver malfunction that is acting as disruption. Reducing the number of available receivers does not introduce any outliers but reduces the number of available variables to localize the source accurately. And since we used only those receivers with very high SNR, we assumed that the TOAs are extracted accurately, which eliminates the factor of arc-tangent performing better than the linear loss function because of wrongly extracted TOAs.

## 7. Conclusions

An end-to-end simulation framework was developed for a sample acoustic indoor localization system, and its usefulness was demonstrated by evaluating and analyzing the robustness and resilience of the system in the presence of disruptions. The framework was used to simulate and assess the behavior of the sample system in the presence of individual disruptions and the combination of disruptions, which allowed us to investigate inter-dependencies of disruptions systematically. The approach enables modeling the expected effects of disruptions present only for a limited time, particularly the localization accuracy loss as soon as the disruption is present (time-dependent response to disruption) and the accuracy gain after it is removed (recovery bounce-back). Such time-dependent assessments are essential for resilience analysis in operational contexts. The resilience of the sample system was also quantified.

It was found that the arc-tangent loss function can drastically improve the system performance in the presence of single disruptions, except in the case of pure receiver malfunction. However, when combining the disruptions (hybrid disruptions) for more realistic disruption scenarios, the performance of the arc-tangent loss function always dominates the performance of the linear loss function. It was also shown that the system is highly resilient to disruptions when using the arc-tangent loss function.

Future work may consist of an evaluation of this scheme for more (acoustically) complex room geometries and multiple acoustic sources including complex situations like multi-path effect and multiple occlusions. Additional disruptions could also be introduced. It is also of great interest to extend this method to simulate moving sources and time-dependent disruptions appearing in time and space to generate true time-dependent behavior, in particular, to generate resilience curves.

## Figures and Tables

**Table 1:** Comparison of localization error between arc-tangent and linear loss functions for single disruptions. From all the plots one single value is extracted for comparison. The extracted values are: Case 2 barrier, SNR = 5.0 dB and 5 active receivers.

Loss Function	Barrier (Figure 5)	Noise (Figure 6)	Receiver Malfunction (Figure 7)
Linear	4.2 m	2.2 m	0.12 m
Arc-tangent	1.9 m	0.2 m	0.12 m

**Table 2:** Top-level comparison of localization error between arc-tangent and linear loss functions for multiple disruptions. From all the plots one single value is extracted for comparison. The extracted values are: Case 2 barrier, SNR = 5.0 dB and 5 active receivers.

Loss Function	Barrier + Noise (Figure 8)	Noise + Receiver Malfunction (Figure 9)	Receiver Malfunction + Noise (Figure 10)
Linear	6.4 m	1.9 m	2.1 m
Arc-tangent	1.8 m	0.2 m	0.2 m

**Table 3:** Resilience quantification of the simulated indoor acoustic localization system for both arc-tangent and linear loss functions.

Loss Function	Resilience (***Rsl***)
Linear	0.634
Arc-tangent	0.936

## Data Availability

The data presented in this study are available on request from the corresponding author.

## Abbreviations

The following abbreviations are used in this manuscript:

ASSIST	acoustic self-calibrating system for indoor smartphone tracking
CPS	cross power spectrum
RFID	radio frequency identification
RIR	room impulse response
SNR	signal-to-noise ratio
TDOA	time difference of arrival
TOA	time of arrival
